# A Review of the Surveillance Techniques for *Aedes albopictus*

**DOI:** 10.4269/ajtmh.20-0781

**Published:** 2022-10-31

**Authors:** Qin-Mei Liu, Zhen-Yu Gong, Zhen Wang

**Affiliations:** Zhejiang Provincial Center for Disease Control and Prevention, Hangzhou, China

## Abstract

*Aedes* (*Stegomyia*) *albopictus* (*Skuse*) (*Diptera: Culicidae*) transmits a variety of arboviruses (arthropod-borne viruses) and acts as one of the most dangerous mosquito species in the world. Mosquito surveillance is the main means of evaluating vector density, vector-borne disease risk, and the efficacy of vector-control operations. The larval density of *Ae. albopictus* can be reflected by means of Breteau index and Route index, and egg density can be monitored by ovitrap and mosq-ovitrap, whereas mosquito surveillance methods mainly include human landing catch, human-baited double net trap, BG-Sentinel trap, autocidal gravid ovitrap, gravid *Aedes* trap, and mosquito magnet. This article describes different methods of *Ae. albopictus* surveillance and offers suggestions to improve surveillance.

## INTRODUCTION

Vector-borne diseases such as dengue, chikungunya, and lymphatic filariasis, pose a major threat to human life and health, especially in tropical and subtropical regions. More than 80% of the global population lives in areas at risk from at least one major vector-borne disease, with more than half at risk from two or more. Vector-borne diseases cause more than 700,000 deaths every year, accounting for about 17% of the global burden of communicable diseases.^1^^,^^2^ They also cause huge economic losses and hinder the development of rural and urban areas. Remarkable progress has been made globally in the fight against malaria and lymphatic filariasis,^1^ but the burden of other vector-borne diseases, especially mosquito-borne diseases, has increased in recent years.^3^ Since 2014, dengue, chikungunya, yellow fever, and other mosquito-borne diseases have been raging in many countries, taking lives and overburdening the health system. In 2016, Zika virus infection and its related complications spread rapidly in the Americas region of the WHO, which had a direct impact on individuals and families and caused social panic and economic loss.^4^

*Aedes *(*Stegomyia*)* albopictus *(*Skuse*) (*Diptera: Culicidae*) (*Ae. albopictus*) is a competent vector for at least 22 arboviruses such as dengue, Zika, yellow fever, and so on.^5^^–^^8^ At present, many *Aedes*-borne diseases have neither vaccines nor special therapeutic drugs. It has been proved that vector control is the most effective measure to prevent transmission of these diseases.^9^^,^^10^ Real-time grasps of accurate *Ae. albopictus* population and density dynamics information is an important part of effective prevention and control.^11^ At the same time, *Ae. albopictus* density surveillance can guide us to carry out timely mosquito control and provide the basis for evaluating the control effect. From a public health perspective, this is important and indispensable.

## LITERATURE SEARCH

Electronic searches were conducted in PubMed, China National Knowledge Infrastructure (CNKI) and Google Scholar with the keywords of “*Aedes*”, “surveillance” and “adult or larva” to identify relevant articles published up until October 21, 2021.

A basic PubMed search was performed to identify commonly used terms in literature describing *Aedes*, surveillance, adult, and larva. Through trial and error, we determined the most comprehensive and relevant literature search field combination, and we also used the Google Scholar search engine and China National Knowledge Infrastructure (CNKI) to obtain relevant information that was not available in PubMed until October 21, 2021. Articles were further preselected based on titles and abstracts, and a thorough assessment of relevance was conducted by full text reading. After the literature search, additional references were added to the author’s file if they were relevant to the topic and helpful for the discussion.

## SURVEILLANCE OF ADULT *AEDES ALBOPICTUS*

*Ae. albopictus* is a day-biting species, which synchronizes its feeding behavior with human activity.^12^ Adult mosquito surveillance is conducted to determine mosquito density, species composition, spatiotemporal distribution, and it can also collect mosquito samples for pathogen screening.^13^ Compared with larval surveillance, it can accurately predict the epidemic trend of the disease in a timely manner.^14^ Adult mosquitoes collected by surveillance can be identified to species level by using morphological keys with reference to “Fauna Sinica, Insecta, Eighth Volume, Diptera, Culicidae”^15^ or “Walter Reed Biosystematics Unit, Systematic Catalog of Culicidae”.^16^

### Human landing catch.

Human landing catch (HLC) is a traditional gold standard method for adult mosquito surveillance.^17^ It is also the most efficient surveillance method for highly anthropophilic mosquito species like *Ae. albopictus*. It is suitable for capturing mosquito species that prefer to feed on human or both human and animal blood. During the hours of peak mosquito biting, human volunteers wearing long clothes and trousers collected mosquitoes landing on their exposed right or left legs using aspirators and torchlights for 30 minutes or more. Because *Ae. albopictus* can be strongly attracted by lactic acid and odor secreted by the human body, some researchers believe that HLC is a sensitive method,^18^ which can be used as a standard reference method to evaluate the surveillance effect of other methods.^19^ In addition, due to its simple operation and low independence of equipment, HLC is suitable for routine *Ae. albopictus* surveillance. However, due to concerns of potential health risks to collectors, especially in epidemic areas, this method is not recommended.^20^^,^^21^ Moreover, another limitation of HLC is that different collectors have different collecting skills and are also attractive to mosquitoes to varying degrees. Before using this method, ethical certification and informed consent are required.

### Human-baited double net trap.

The human-baited double net trap (HDN) consists of two box nets, the inner net falling to the ground protects the human bait, and the outer net is raised 35 cm off the ground, so that mosquitoes lured to the human-bait could fly into the space between the nets. The human bait exposes two legs in the inner net, and another collector collects mosquitoes between the nets using a mosquito respirator, who is protected by long closes or repellent. To a certain extent, the HDN can effectively protect the monitoring participants.^17^ As a common method for dengue vector surveillance, it is relatively economic and efficient. At the same time, China has taken its bite index as an important exponent of dengue control.^22^ In Lao PDR, four methods including HND, carbon dioxide–baited light trap catch (CB-LTC), BG-Sentinel (BGS) trap, and Suna trap were compared; the HDN is more effective than the other three methods in both day and night.^23^ It can be used as a more ethical alternative to HLC for *Aedes* sampling.^17^

However, HDN underestimates the density of *Ae. albopictus* compared with HLC because of its two-layer-nets design, which could result in a lower surveillance result.^24^ HDN also has some shortcomings, such as the need to set up tents on the spot; the operation is relatively complex and needs two participants acting as bait and catcher.^17^ The samples obtained by HLC and HDN can be used to evaluate the resistance of the mosquito population.

### BGS trap.

The BGS trap is designed with a combination of attractive visual and olfactory cues. It is collapsible and portable. The BGS trap is essentially a collapsible fabric container with a white lid with holes covering its opening. It is 36 cm in diameter and 40 cm high. Its attractant simulates the odor produced by the host, which is composed of lactic acid, ammonia and acetic acid or with CO_2_.^25^^,^^26^ The BGS trap has become the industry standard because of its good collection effect on *Aedes* collection.^27^^–^^29^ The lure is strongly recommended,^30^ and without the need for carbon dioxide, it is superior.^31^^,^^32^ Meanwhile, the synergistic effect of CO_2_ and the BG lure makes the most efficient combination in attracting *Ae. albopictus*.^33^

BGS-trap has been extensively field-tested in many countries, and it has been proved as a good tool for the *Aedes* collection. In two Italian cities, mosquitoes were collected with HLC and BGS traps without CO_2_, with the similar numbers of *Ae. albopictus* females during 0.5 or 1.5 hours.^34^ In addition, CO_2_ is also to be an effective mosquito attractant at long and medium distances. The BGS trap is a tool used for routine mosquito surveillance of *Ae. albopictus* in America, when used with lure and CO_2_, the BGS trap collected 33 times more females than CB-LTC per 24 hours.^35^ Australia Darwin port intercept the exotic *Ae. albopictus* by BGS-trap with CO_2_.^36^ In another study carried out on La Réunion Island, Indonesia, researchers confirmed that a mouse-baited BGS trap provided an efficient tool for trapping.^37^ In New Jersey, a study reported that BGS trap placement had a significant effect on *Ae. albopictus* capture rate, and traps in shade or partial shade locations captured 3 times more insects than sunny locations.^38^ Moreover, the operation of the BGS trap is simpler and more standardized than HDN and HLC, and its results are not easily influenced by monitoring personnel. The BGS trap with CO_2_ accounted for more than 85% of all *Ae. albopictus* captured and was significantly more effective at detecting the presence of *Ae. albopictus* compared with the other three techniques (Gravid trap, HLC, and Aspirator).^39^

In most cases, the BGS trap has shown high efficiency in *Ae. albopictus* sampling; however, there were also inconsistencies in some cases. In a study carried out in Yunnan Province of China, 23 BGS traps were placed in the field to catch mosquitoes for 328 hours; only eight *Ae. albopictus* were caught.^40^ Moreover, some researchers found that BGS traps collected insufficient numbers of female mosquitoes for pathogen isolation and that there were construction quality issues.^38^ As a new mosquito surveillance device in some regions, the BGS trap has a high price, and its operation needs a power supply,^41^ so it is difficult to popularize in some economically backward areas.

### Autocidal gravid ovitrap.

The autocidal gravid ovitrap (AGO) consists of three primary components: a black pail that contains hay and water to attract ovipositing female *Aedes* mosquitoes; a capture chamber is attached to the bucket, with a net cover that allows mosquitoes to enter, and on the bottom, a fine mesh prevents mosquitoes from reaching the water; and there is a sticky lining inside the chamber to which mosquitoes adhere.^42^ AGO is mainly used for surveillance and control of the dengue vector *Aedes* (*Stegomyia*) *aegypti* (L.),^41^^–^^44^ and it can also be applied for *Ae. albopictus*.^6^^,^^7^^,^^45^

Field trials in Puerto Rico demonstrated that AGO captured more *Ae. aegypti* gravid females and provided higher sensitivity than conventional ovitraps.^44^ Moreover, there was a significant positive correlation between mosquito captures using the BGS trap and AGOs, indicating that AGOs are useful and low-cost mosquito surveillance devices.^41^ A study reported that a modified AGO with a powered suction fan or additional lures (BG lure or BG lure + octenol)^46^ could increase collections of *Ae. aegypti* and *Ae. albopictus*.^47^ Compared with clean water, hay infusion (or Bermuda grass infusion),^48^ larval rearing water, solution of ammonium phosphate and potassium nitrate are more attractive to gravid female *Ae. albopictus*.^49^^,^^50^ Furthermore, raising the size of the trap entrance, changing the white color of trap components to black, and adding the volume surface area of the aqueous bait could significantly improve the performance of the AGO.^44^

### Gravid *Aedes* Trap.

The Gravid *Aedes* Trap (GAT) consists of a 10-L black bucket base, a translucent top chamber, a black nylon mesh placed between the translucent chamber and base, and a black plastic entrance funnel. The black entrance funnel (12 cm in diameter) was inserted on the top of the translucent chamber and extended 6.5 cm into the GAT top.^51^ The GAT is a passive trap that relies on visual and olfactory cues to lure and capture gravid mosquitoes.^52^ When mosquitoes enter the transparent chamber through the black funnel on top of the trap, because the black mesh net provides a barrier between the mosquito and the infused water, it is difficult for the mosquito to escape from the transparent chamber. At the same time, they are exposed to a sticky surface,^53^ oil,^54^ or insecticides.^51^^,^^55^

The black color of GAT attracts mosquitoes from afar. Therefore, the trap needs to be placed where it is readily visible but protected from rain. In field trials of northern Australia, GAT collected 2 to 4 times more female *Ae. aegypti* than the MosquiTRAP and the double sticky ovitrap.^55^ A study in Northeastern Florida also proved the GAT a highly effective surveillance tool, it collected overall 6-fold more *Ae. albopictus* than the AGO.^56^ Moreover, it can be used effectively both indoors and outdoors. It is important that GAT can incorporate a noninsecticide killing agent without reducing collections.

Although the researcher suggested that it underperforms compared with the BGS trap,^54^ the GAT could still be used as an additional tool to reduce an adult *Ae. albopictus* population. To achieve a better performance, sufficient traps must be deposited at a suitable distance of approximately 25 m or less.^57^

### Mosquito magnet.

The principle of Mosquito Magnet (MM) is to use liquefied petroleum gas to produce a certain concentration of CO_2_, heat, and water vapor through catalytic action, and at the same time use negative pressure technology to lure and trap mosquitoes.^58^^–^^61^ There are many types of MM, such as the P-type, L-type, and X-type.^62^

In a large tire repository, seven traps (MML, Fay-Prince, Dragonfly, moving-target trap, CDC without light, Center for Disease Control and Prevention light trap catch (CDC-LTC), Mosquito Deleto) were tested, significantly higher mean numbers of *Ae. albopitus* and *Ae. aegypti* females were collected with the MM compared with the remaining traps.^63^ There was also a study that showed the trap effect of MMP was significantly better than CDC-LTC and CB-LTC. Compared with the BGS trap, the capture rate of MM is 3 to 10 times greater than that of the BGS trap. MM also performed better than the BGS trap under a range of meteorological conditions (rain and wind).^65^

Compared with the basic version of MM, the addition of octenol,^66^ lactic acid, ammonia + lactic^61^ acid or octenol + lactic acid^67^ can improve the capture rate of *Ae. albopictus*. It has the advantages of labor-saving, little influence from human factors, and does not require an external power supply.^58^ Its waterproof design^68^ guarantees good performance under a variety of weather conditions.^65^ However, MM also has disadvantages, such as its high cost and importability. In some studies, MMX collected fewer *Ae. albopictus* compared with Mos-Hole and the BGS trap.^69^ The samples obtained by AGO, GAT, and MM can be used for vector-borne pathogen detection and population estimation.

## LARVAL SURVEILLANCE OF *AEDES ALBOPICTUS*

*Ae. albopictus* is a semidomestic and container-breeding mosquito species,^6^ and its habitat is relatively wide and scattered.^70^ *Ae. albopictus* can breed in indoor and outdoor artificial small- and medium-sized container water,^71^ whereas adult *Ae. albopictus* mainly inhabits the dark and hidden place of the field environment,^72^^,^^73^ such as the forest or bamboo, bush or grass, and waste tires near the breeding place.^74^ The stage of *Ae. albopictus* larvae generally last for 4 to 10 days, depending on water temperature and food richness, and then developed into the pupal stage. Larval surveillance involves sampling a wide range of aquatic habitats for the presence of immature mosquitoes.^13^ It should be noted if there are cryptic aquatic habitats that cannot be visually detected, the surveillance may underestimate the prevalence and abundance of container *Aedes*.

As an important component of *Aedes* surveillance, larval surveillance allows us to identify and treat breeding sites; however, it is often ignored. Its main functions are as follows: to provide us cues of locations suitable for nonchemical treatment, such as breeding sites cleaning and biological treatment; to provide accurate background data of breeding status in regions to be controlled; to evaluate the application and control effect of insecticides on a continuous basis; to provide data of species distribution, density, and seasonal dynamics; to enhance understanding of adult mosquito surveillance; and to provide an evaluation of resistance surveillance.^21^

### Larvae pipette method—Breteau index.

The larvae pipette method is used to monitor the density of mosquito larvae and pupae in various environments, mainly to check whether there are larvae and pupae in the container, as well as the species and quantity. The indicators include the 100-househoid index, larval density index, container index (CI), and house index (HI). One hundred-household index is defined as number of positive containers per 100 houses inspected. It is also known as the Breteau index (BI) for monitoring the larvae and pupae of *Ae. albopictus* or *Ae. aegypti*.^75^^,^^76^ It can objectively reflect the larval dynamics in the natural environment, providing data on species richness, composition, and distribution characteristics of *Aedes* breeding place.^77^ Before the surveillance, the residents should be publicized and educated to improve their understanding of the hazards of the *Aedes* mosquito, which could act as biting harassment and vectors of many deadly diseases, and further increase public awareness of the importance of turning over pots and pouring cans to remove mosquito breeding sources.^78^^,^^79^ It was aimed to give “help for self-help” and to transform the public from “spectators” to “actors.”^80^

BI is an important index to evaluate *Aedes* density and risk of community transmission during the dengue epidemic.^81^ It is easy to understand, and there are several risk assessment thresholds: BI < 5 (low infestation and low risk of dengue transmission); BI ≥ 5 (the risk of transmission); BI ≥ 10 (the risk of the outbreak); BI ≥ 20 (the risk of the regional epidemic).^22^ However, these values need to be verified when applied to dengue fever transmission, and the critical value should be adjusted according to the purpose, manpower and material resources to get more reasonable sensitivity and specificity in practice. A study carried out in Guangzhou, China, predicted that the BI thresholds of dengue transmission and outbreak could be determined as 5.0 and 9.5 respectively,^82^ according to the study of BI and dengue cases in 2006–2012. Some researchers believed that the most direct operational indicator for predicting the transmission risk of dengue is BI > 4.^81^^,^^83^

### Path distance method-Route index.

Route index (RI) refers to the number of positive containers per kilometer of inspection route.^21^ Path distance method is suitable for monitoring small-size container water with larvae and pupae breeding in the field and is easy to operate. The RI can be used to evaluate the breeding status and mosquito control effect in a city and then establish and consolidate the achievement of a national health city in the future.

One study suggests that values of RI and BI are consistent, and there is a strong positive correlation between the two indices.^84^ During 2006–2012, a field study was conducted in Guangzhou, China, to evaluate *Ae. albopictus* breeding status using both RI and BI. The study suggested that the number of positive habitats reported in Guangzhou City was underestimated, compared with RI.^85^ In the urban environment or during the dengue emergency, the path distance method is much easier to operate and RI can serve as an effective supplement to BI.^86^ Oruxmaps is a free GPS software, and it can serve as a tool for an accurate distance calculation of path distance method, which could effectively reduce false behavior and then ensure the surveillance quality. The samples collected by larvae pipette method and path distance method can be used to detect mosquito-borne pathogens and evaluate insecticide resistance.

## EGG SURVEILLANCE OF *AEDES ALBOPICTUS*

Oviposition traps are artificial containers baited with an infusion that acts as an attractant. Those traps are widely used to sample aedine mosquitoes such as *Ae. albopictus*. Ovitrap was developed based on the premise that gravid females must look for oviposition sites.^13^ The oviposition behavior of gravid females involves two crucial events. The first is to use remote cues such as color, texture and chemicals to find suitable habitat, and the second is the short-range cues that gravid females use to determine to lay eggs in the habitats, such as disturbance, the chemical properties of the water, and presence of conspecific individuals or other organisms.^72^

It should be noted that for egg surveillance in areas with more than one species of *Aedes*, eggs need to be hatched and larvae reared to be able to identify the species.

### Oviposition trap (Ovitrap).

An ovitrap (OT) is a small plastic container, usually dark in color, containing water and a substrate where female mosquitoes lay their eggs.^87^ OTs are usually used to collect *Aedes* eggs.^88^ Its data indicate that it is suitable for assessing the presence of *Ae. albopictus* at a given site, but not adult abundance.^89^ At low population density, OT also can detect *Ae. albopictus*.^90^ Moreover, the number of eggs in OT is usually regarded as the only indicator of high nuisance or high risk of disease transmission and is used to plan mosquito control operations.^91^ An OT has the advantages of being sensitive, easy and inexpensive to construct, portable, and does not require electricity or carbon dioxide, and not invasive, so it can be extensively used as an effective tool for *Aedes* egg surveillance on site.^92^

Field tests proved that germination paper was the most appropriate oviposition substrate, and hay, pennisetum grass hay, or rice straw infusion could increase egg collecteds.^93^^,^^94^ Compared with tap water, water from natural environment ponding that containing or previously contained conspecific larvae^95^^,^^96^ or dead grass leaching would be more attractive^97^ because there are leaves, dead grass and other substances produced after degradation, such as plankton, which are more suitable for the growth of larva. It is suggested that infusions made using a wider range of plant biomass and over a longer fermentation period could vigorously attract *Ae. albopictus*.^98^ Moreover, a mixture of multiple synthetic materials based on n-heneicosane could also be used as an attractant.^12^

### Mosquito-oviposition trap.

The mosquito-oviposition trap (MOT) was first described by Lin et al.^99^ This device consists of a transparent cylindrical plastic jar with a concave bottom and a black top cover with three conical holes. A white circular filter paper can be placed inside the bottom of the jar as an oviposition substrate, and it can collect both *Aedes* eggs and adults. The MOT has been widely used in routine surveillance of *Ae. albopictus* in China.^100^ It is designed by reference of *Aedes*’ oviposition preference of small-size containers (especially black^101^) and the conical holes in the cover are designed for easy entry but difficult exit for the mosquitoes, which is similar to the structure characteristics of the fly trap.^99^

As a modified OT, MOT is also cheap, portable, and sensitive in detecting *Aedes* mosquitoes; moreover, this device can also collect adult *Aedes* mosquitoes, which could meet the technical requirement of specimen collections for mosquito-borne pathogens screening.^102^ In urban environments, the stability and sensitivity of the MOT are higher than that of BI^103^; in rural areas, the sensitivity of MOT is higher than that of CI.^99^ Because MOT is also suitable for areas with low *Aedes* density or few breeding sites,^104^ MOT could be a good choice for *Aedes* surveillance when risk assessment is needed after dengue-related emergency mosquito control.^105^

MOTs have certain shortcomings. Data are unable to predict differences of *Ae. albopictus* population abundance among different locations. There were studies indicating that the efficiency of trapping adult mosquitoes was not significantly lower that of eggs collection.^102^^,^^106^ Additionally, the surveillance period is fairly long, and the sampling result is greatly affected by monitoring the environment, weather, and other factors. The samples obtained by BGS-trap, OT, and MOT can be used for virus detection, insecticide resistance, and population estimation.

## OTHER SURVEILLANCE METHODS

In addition, there are many monitoring methods, such as BG-counter, BG-bowl, MOS-hole, and smart trap (Korea), and In2Care traps, sweep net, citizen science, modeling, that are not described in this article.

## CONCLUSIONS

Different species of mosquitoes have various ecological habits, so when monitoring mosquito species, each surveillance method has advantages and disadvantages, as long as the corresponding surveillance method is selected according to the needs, the results will be optimal. The most important role of vector surveillance is to control the science-based evidence, effectiveness of results, the real-time reporting of data and then adjust the strategies and methods of control according to the evaluation report. We then take integrated environmental control, chemical control, biological control, and other measures to form a set of systematic prevention and treatment plan to keep mosquitoes damages under control and thus prevent the occurrence of mosquito-borne diseases and improve public health.
